# Pioneering Experience of First Kidney Paired Donation in Brazil

**DOI:** 10.1590/2175-8239-JBN-2021-0259

**Published:** 2022-02-02

**Authors:** David José de Barros Machado, William Carlos Nahas, Elias David

**Affiliations:** 1 Universidade de São Paulo, Hospital das Clínicas da Faculdade de Medicina, São Paulo, SP, Brasil.

Dear editor,

The supply of kidneys from deceased and living donors in Brazil cannot meet the growing annual demand for transplants^1^. ABO incompatibility (ABOi) or positive complement-dependent cytotoxicity (CDC) crossmatch between the candidate recipient and the prospective donor is a major obstacle to living donor kidney transplant, and consequently many recipients remain on the waiting list for years^2^. Based on a simulated model, Bastos et al. recently showed that with the implementation of the Kidney Paired Donation (KPD) program in Brazil, the number of transplants with living donors could increase by 23%, using an optimized algorithm and the number of sensitized recipients transplanted could increase by 70.7% when using the prioritizing model^3^. In KPD, a potential kidney recipient that has a willing but incompatible living donor receives a kidney from the donor of another incompatible pair and vice versa.

Since 2011, we have started the discussion and preparation for the implementation of a local KPD program at the Hospital das Clínicas - FMUSP. We discussed the topic internally with our Bioethics Commission, Medical Ethics Commission, Organ and Tissue Transplant Commission, and Clinical Board. In Brazil, Law No. 9,434 regulates the removal of organs, tissues, and parts of the human body for transplant purposes, without contemplating the activity of paired donation. Therefore, we proposed and obtained ethical approval (CAAE 83469418400000068) for a research project to assess the effectiveness of transplantation in patients with ABOi living donor or positive CDC/flow cytometry crossmatch by donor exchange. At the end of the medical, psychological, social, and immunological evaluation of the pairs and after the consent terms were signed, approval was obtained from the Ethics Committee that evaluates transplants with unrelated donors to perform the paired donation. Subsequently, we also obtained the approvals of the Public Prosecutor’s Office, the judicial approval and that of the General Coordination of the National Transplant System - Ministry of Health, for the first transplant with KPD in Brazil.

On March 10, 2020, the 38-year-old recipient (recipient 1) with chronic glomerulonephritis, undergoing dialysis treatment, on a deceased transplant waiting list for 8 years and with the previous exclusion of 7 living donors, including his wife (donor 1), because of ABOi, received the kidney of the 45-year-old donor of the second pair. The second recipient, 57 years old, with chronic glomerulonephritis, undergoing dialysis treatment, on a deceased transplant waiting list for 1.9 years, and with exclusion of his only donor, wife (donor 2) because of ABOi, received the kidney from the 39-year-old donor of the first pair (donor 1). Pair anonymity was assured until the time of admission, as was reciprocal compatibility between the pairs and simultaneous surgery in 4 operating rooms, which allowed donors to withdraw consent at any time before anesthesia. After 12 months, recipients had adequate kidney function and their donors were doing well.

Efforts to increase organ supply have a significant source of potential donors in the paired donation programs. Today, all countries that are world leaders in transplantation are practicing and developing this modality in an attempt to reach more and more recipients because of its excellent results^2,5^. We expect that this HCFMUSP program provide the impetus for the national development of DRP.
